# Robotic-Assisted Laparoscopic Radical Prostatectomy: An Objective Assessment and Review of the Literature

**DOI:** 10.1100/tsw.2006.400

**Published:** 2006-08-07

**Authors:** Lester S. Borden Jr., Paul M. Kozlowski

**Affiliations:** Section of Urology and Renal Transplantation, Virginia Mason Medical Center, Seattle, WA, USA

**Keywords:** robotic prostatectomy, laparoscopic prostatectomy, radical prostatectomy, prostate cancer, review

## Abstract

Robotic-assisted laparoscopic radical prostatectomy (RLRP) has become an accepted treatment option for men with prostate cancer. A search of the available literature through January 2006 was performed to analyze the surgical technique, outcomes data, and other unique issues regarding RLRP. While prospective, randomized trials and long-term data are lacking, short-term data from single institution series have demonstrated outcomes for RLRP that appear to be equivalent to those for open radical prostatectomy (ORP). Although not yet proven, some encouraging data suggest that RLRP may be able to achieve improved cancer control, postoperative urinary control, and erectile function compared to open surgery for prostate cancer. Definite advantages of RLRP over ORP are not yet established. Future studies will determine the role of RLRP in the surgical treatment of men with prostate cancer.

### INTRODUCTION

Prostate cancer is the most common noncutaneous malignancy of men in the U.S. An estimated 232,090 new cases were predicted to occur in the U.S. in 2005 with 30,350 patients expected to die from their disease[1]. Radical prostatectomy is an effective treatment for organ-confined prostate cancer with excellent long-term cancer control and recent evidence demonstrating a survival benefit for patients with localized disease[2,3,4,5,6,7]. Due to the substantial life expectancy following surgery, quality of life after therapy is consequently of great importance when discussing treatment options. Laparoscopic and robotic-assisted laparoscopic radical prostatectomy (RLRP) techniques have been introduced in an attempt to decrease the morbidity associated with open surgery. Initially, the goals of these minimally invasive techniques were to decrease postoperative pain, lessen blood loss, shorten convalescence, and improve cosmesis. Additionally, it was hoped that these new techniques would improve postoperative urinary and erectile functional outcomes after surgery.

Laparoscopic radical prostatectomy (LRP) was first reported in the early 1990s with reports of RLRP soon following[8,9,10]. The steep learning curve and advanced laparoscopic skills required to efficiently perform LRP have been a major hindrance to the widespread acceptance of this technique in the U.S.. In contrast, RLRP requires a much shorter learning curve, allowing surgeons with limited laparoscopic experience to offer their patients the benefits of minimally invasive surgery. RLRP also offers certain technological advantages over LRP and open radical prostatectomy (ORP) including three-dimensional (3-D) viewing with improved magnification, elimination of surgeon tremor through motion-scaling technology, and specialized instrumentation that facilitates fine, precise dissection. These factors have contributed to the recent popularization of RLRP.

Despite theoretical advantages of RLRP over ORP, actual improvements in terms of oncologic outcome or morbidity are currently unproven and controversial. This review examines the current available literature analyzing the surgical technique, outcomes data, and other unique issues involving RLRP.

### TECHNOLOGY

Currently, there is only one robotic system in use that is widely available (daVinci®, Intuitive Surgical, Sunnyvale, CA). Originally developed to enable the performance of trauma surgery on the battlefield from a safe and distant location, the device utilized is actually a master-slave system that is directly controlled by the surgeon at a remote console. This system involves three main components: a vision cart with a specialized vision system that provides 3-D viewing for the surgeon, a patient-side surgical cart responsible for physically moving surgical instruments within the patient, and a surgical console where the operating surgeon directly controls the movements of the surgical cart (Fig. 1). The surgeon uses intuitive finger-controlled movements (similar to what he or she would use in open surgery) to control the instruments while sitting at the remote console. Using specialized computer software, the surgeon's hand movements are transferred exactly and without delay to the microsurgical instruments at the operative site. Surgeon tremor may be reduced or eliminated by selecting options on the console that allow surgeon movement to be filtered on a scale of 1:3 or 1:5. There are a variety of instruments that may be used and can be exchanged via 8-mm ports by a patient-side assistant during the course of the operation. These instruments have the ability to perform wristed movements with seven degrees of freedom to allow precise dissection in the limited confines of the human pelvis.

There are some drawbacks of robotic-assisted technology. One disadvantage is the absence of tactile feedback, present in both open and to some extent in conventional laparoscopic surgery. This obligates the surgeon to rely completely on visual feedback to perform the operation. Another obstacle to the widespread utilization of RLRP has been the high cost associated with purchase and maintenance of this technology.

### SURGICAL INDICATIONS

The goal of RLRP is identical to that of ORP. While slight differences in the approach may exist (i.e., antegrade vs. retrograde dissection, intraperitoneal vs. extraperitoneal), the end result is the same — removal of the prostate and seminal vesicles. Indications for performing RLRP are also identical to ORP — clinically localized prostate cancer in men with a life expectancy of greater than 10 years. Some relative contraindications specific to RLRP include prior intra-abdominal surgery, patient obesity, or large prostate size. With increased experience, each of these relative contraindications is much less of a factor.

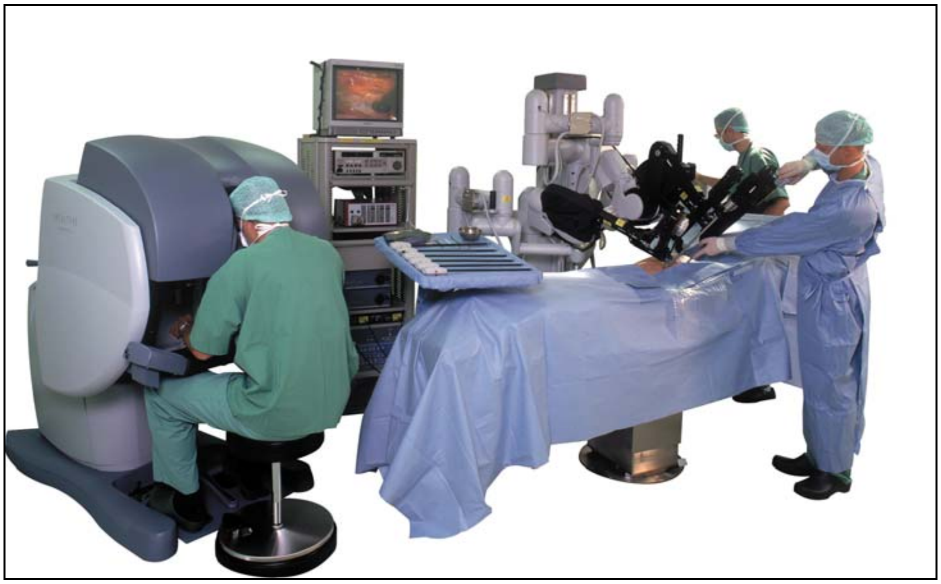

**FIGURE 1**. The daVinci® robotic system: surgical console, vision cart, and patient-side surgical cart. Photo courtesy of Intuitive Surgical, Sunnyvale, CA.

### TECHNICAL CONSIDERATIONS

In general, RLRP is a hybrid procedure that combines aspects of both the LRP and ORP technique. Multiple authors have described their technique for performing RLRP[11,12,13]. A brief outline of the steps involved during RLRP is provided (Table 1). While surgeons have individual preferences regarding technique, a few specific issues related to the technical aspects of the procedure deserve mention and are discussed below.

#### Surgical Approach

In contrast to open retropubic radical prostatectomy, most surgeons use an intraperitoneal approach for RLRP. Theoretically, a transperitoneal approach can result in a higher incidence of postoperative ileus compared to an extraperitoneal approach. This may be caused by bowel manipulation or urine extravasation within the intraperitoneal space. A transperitoneal approach also introduces a risk of inadvertent intraperitoneal bowel injury that is virtually nonexistent with ORP. Postoperative hematomas and urine leaks may also be managed more easily when an extraperitoneal approach is utilized. The feasibility of extraperitoneal RLRP has been explored and successfully demonstrated[14]. Some surgeons have advocated this approach for the previously mentioned reasons. Still, most RLRP surgeons currently favor a transperitoneal approach due to the larger working space that allows the robotic instruments to maximize their full range of motion and overall low risk of complications.

#### Patient Selection

Obesity increases the difficulty of radical retropubic prostatectomy surgery and may partly explain the inferior outcomes for these patients[15]. Ahlering et al. examined the impact of obesity on RLRP outcomes and found that longer OR times, increased blood loss, and longer hospital stays were more common in obese patients[16]. In addition, obesity increased the risk of postoperative complications and resulted in longer times required to regain urinary control and a return to baseline activity levels.

**TABLE 1**
**Technical Steps of RLRP**

| | |
|---|---|
| 1. | Patient positioning |
| 2. | Port placement |
| 3. | Docking of surgical cart |
| 4. | Mobilization of bladder and exposure of prostate |
| 5. | Incision of endopelvic fascia |
| 6. | Ligation of dorsal venous complex |
| 7. | Bladder neck transection from prostate |
| 8. | Dissection of vasa deferentia and seminal vesicles |
| 9. | Establishment of plane between prostate and rectum |
| 10. | Control of prostatic pedicles |
| 11. | Preservation of neurovascular bundles (if indicated) |
| 12. | Transection of urethra |
| 13. | Pelvic lymphadenectomy (if indicated) |
| 14. | Vesicourethral anastomosis and placement of new catheter |
| 15. | Retrieval of prostate specimen |
| 16. | Placement of drain |
| 17. | Closure of port sites |

Anatomic variations can also increase the difficulty and complexity of RLRP surgery. Adequate working space is necessary to allow maximal function of the robotic instrumentation within the pelvis. As such, RLRP can be more difficult in a patient with a particularly narrow pelvis. Large prostates may also result in a more challenging procedure due to space limitation. Large glands limit the ability to manipulate the prostate within the pelvis, which impairs the ability to create countertension necessary during dissection of the prostate. Prostates with a large median lobe component also increase the technical difficulty of RLRP due to the distortion of normal anatomy that may be present. With experience, each of these technical challenges can be overcome and rarely necessitate conversion to an open procedure.

### Surgical Assistance

RLRP does require an additional person to provide laparoscopic assistance at the patient's side during the course of the procedure. The patient-side assistant exchanges the robotic instruments, passes suture in and out of the body, and manipulates the Foley catheter to aid performance of the urethral anastomosis. In addition, every step of the operation can be facilitated by subtle maneuvers that create countertension during the prostate dissection and by maintaining a bloodless field with meticulous suction. While a physician assistant or nurse may serve as the surgical assistant, a skilled laparoscopic surgeon may shorten the learning curve for new robotic surgeons and may provide superior oncologic and functional outcomes.

### PRIMARY OUTCOMES

The most important outcomes for an individual patient following surgical treatment of prostate cancer are cancer control, urinary control, and erectile function. While cancer control is usually the primary goal for patients, quality of life issues are also extremely important as most men have a substantial life expectancy following surgery. Furthermore, as oncologic outcomes improve, urinary and erectile functional outcomes become a more important priority for patients when deciding on treatment. Results regarding these outcome measures are available from large ORP series and should be considered the gold standard against which to compare RLRP.

### Cancer Control

Radical prostatectomy provides excellent long-term cancer control in patients with prostate cancer. Fifteen-year, cancer-specific survival rates of 80–90% are reported by multiple large series[3,4,5]. The most compelling data to support the effectiveness of radical prostatectomy comes from a Scandinavian randomized trial that demonstrated a significant benefit in terms of progression to metastatic disease and survival when compared to watchful waiting[2]. While no endpoint is an adequate surrogate for survival data, other factors shown to be associated with survival are used in studies with shorter follow-up. Two common endpoints used to assess oncologic control in cohorts with short-term follow-up are positive surgical margin rate and prostate-specific antigen (PSA) recurrence.

Positive surgical margins have been demonstrated to confer an adverse prognosis for patients who have undergone radical prostatectomy[17,18]. Many factors may affect the positive margin rates including surgeon experience, method of pathologic analysis, and the volume/extent of disease within the prostate. Some of these factors may vary based on patient selection and must be considered when comparing different series. Overall positive margin rates in RLRP series range between 6–30% with rates for organ-confined tumors ranging from 4.5–8%[19,20,21,22,23,24,25]. These rates are comparable to the rates of 6–41% reported by experienced surgeons in large ORP series[4,6,26,27,28]. Some investigators have prospectively compared their experience with both ORP and RLRP. Tewari et al. compared their positive surgical margin rate between 200 RLRPs and 100 ORPs, and found significantly lower positive margins in the RLRP group (9 vs. 23%, $p < 0.05$)[22]. These results should be interpreted with caution since only one surgeon performed all RLRP procedures, while one of eight different surgeons performed the ORPs. Ahlering et al. compared their positive surgical margin rates for RLRP (n = 60) and ORP (n = 60) in 120 cases by the same surgeon and found no significant difference (16.7 vs. 20%, respectively) between surgical approaches[19].

Since the natural history of prostate cancer allows for relatively long-term survival even without treatment, follow-up after therapy usually requires at least 10–15 years to demonstrate a survival benefit. PSA recurrence rate is used as a surrogate endpoint in studies with shorter follow-up. While limited data regarding biochemical recurrence after RLRP are present in the literature, the available information is encouraging. Patel et al. reported 95% of patients with no PSA recurrence at a mean follow-up of 9.7 months[20]. Similarly, others have reported short-term PSA recurrence-free rates over 90% with no difference when prospectively compared to their ORP patients[19,22].

In principle, RLRP is the same operation as ORP. The same structures are removed and there is no logical reason to suspect that RLRP should be an inferior cancer operation in the hands of an experienced radical prostatectomist. As early data demonstrate similar positive margin rates and PSA recurrence rates from RLRP and ORP series, RLRP is likely at least equivalent to ORP in terms of cancer control. With improved visualization and the ability to perform more precise dissection, there is hope that future studies may show improved oncologic outcomes in men undergoing RLRP. In the meantime, it is important to remember that no randomized trials exist that compare RLRP with ORP, and long-term data on cancer control after RLRP is lacking.

### Urinary Control

The definition of incontinence after radical prostatectomy varies from series to series. Most investigators define postoperative continence as requiring no pads to protect against urinary leakage. The return of urinary control may take 12 months or longer and therefore length of time from surgery is an important consideration when comparing series. Large ORP series have reported that up to 93% of patients are continent after surgery[29,30]. Despite these excellent results with ORP, the superior visualization of the urethra during RLRP led many to believe that postoperative urinary control could still be improved.

Two series have thus far reported their continence rates with at least 6 months of follow-up after RLRP. Menon et al. reported 96% of their patients required no pads at 6 months follow-up[21]. They also reported that urinary control returned quicker after RLRP with a median time to continence of 44 days compared to 160 days in their ORP patients ($p < 0.05$)[22]. Patel et al.[20] found that 98% of their patients at 1-year follow-up required no pads, lending further evidence that RLRP appears at least equal to ORP in terms of postoperative urinary control.

### Erectile Function

There are multiple factors to consider when critically assessing reports of erectile function after radical prostatectomy. Similar to reports of postoperative continence, definitions of potency after surgery vary and should be carefully assessed prior to comparing different series. Validated questionnaires should be used and a third party should collect data to maintain objectivity and minimize physician bias. Preoperative function, patient age, and whether a unilateral or bilateral nerve-sparing procedure is performed affect potency results and should be reported. In addition, length of follow-up is important as erectile function may take up to 18 months after surgery to return. For all of these reasons, data from RLRP series are difficult to compare to ORP series.

The highest reported rates of postoperative potency in contemporary ORP series range from 76–86% for patients who are potent prior to surgery and undergo a bilateral nerve-sparing procedure[29,30]. Most other studies report much lower rates. In older men, those with preoperative erectile dysfunction, or in patients who do not undergo bilateral nerve-sparing procedures, the likelihood of postoperative erectile dysfunction increases[29,30,31].

Currently, data regarding potency after RLRP is limited due to short-term follow-up in most series. While evidence suggests similar postoperative potency rates after RLRP and ORP, it has been hypothesized that RLRP could improve postoperative erectile function due to the ability to perform a more precise dissection of the neurovascular bundles[32]. Some early data have supported this theory. Menon et al. were the first to report their post-RLRP potency data: 68% of men under age 60 and 38% of men older than 60 had sufficient erections for sexual intercourse after surgery[21]. More recently, this same group prospectively compared two techniques of intraoperative nerve sparing in men with normal preoperative erectile function defined as having an International Index of Erectile Function (IIEF-5) score > 21[33]. Using a "prostatic fascia–sparing" technique, the authors reported significantly improved potency compared to conventional nerve-sparing technique (control group). At 12 months follow-up, 97% of the study group achieved erections sufficient for intercourse compared to 74% in the control group ($p = 0.002$). Using stricter criteria of a postoperative IIEF-5 score of > 21, 86% of the study group vs. 26% of the control group was potent with or without the aid of phosphodiesterase-5 inhibitor therapy ($p < 0.0001$). The authors attributed their impressive results to the sparing of additional nerves or blood supply within the prostatic fascia that may be important for erectile function. As of yet, these results have not been duplicated by other investigators. Until other authors corroborate these results, RLRP is not considered to be superior to ORP in terms of postoperative potency.

Some men are not candidates for nerve-sparing surgery based on the extent of their prostate cancer. Sural nerve grafting at the time of prostate resection is one option to potentially improve postoperative potency rates in men who have one or both neurovascular bundles resected during radical prostatectomy. The feasibility of sural nerve grafting during RLRP has been demonstrated[34]. Data regarding potency outcomes after sural nerve grafting during RLRP have not yet been reported.

## SECONDARY OUTCOMES

### Blood Loss

Radical prostatectomy has traditionally been associated with significant intraoperative blood loss. Contemporary ORP series report mean estimated blood loss (EBL) to range from approximately 500–900 cc[28,35,36]. There is little controversy that RLRP results in decreased blood loss compared to ORP. Most RLRP series report EBL to be < 250 cc with transfusion rates of 0–6%[19,20,21,22,23,24,25,37]. Menon et al.[22] and Ahlering et al.[19] each found EBL to be significantly less when compared to their open cohorts. Improved visualization, more precise dissection, and the presence of a pneumoperitoneum help to reduce blood loss during the procedure.

Despite less blood loss, many would consider lower EBL a relatively meaningless outcome if blood transfusion rates are no different. Blood transfusion is now unusual after ORP with rates of 2.4–4.6%[35,36,38]. Some would therefore argue that decreased blood loss associated with RLRP is not a significant advantage over open surgery. However, a valid counterargument is that the improved visualization that results from a “bloodless” operative field may theoretically allow more precise dissection which may translate into lower positive surgical margins and improved oncologic control.

### Operative Time

Operative times reported for RLRP vary (Table 2). While RLRP usually takes longer to perform during the initial learning phase, times can eventually be decreased to those of ORP. Ahlering et al. reported that after their initial learning curve, operative times for RLRP and ORP performed by the same surgeon were not significantly different[19].

**TABLE 2**
**RLRP Series**

| Authors | Cases | EBL (Tx) | OR Time | Compl. | LOS | Catheter | Continence | Potency | PMR | PMR for T2 Tumors | BFS |
|---|---|---|---|---|---|---|---|---|---|---|---|
| Ahlering et al. | 60 | 103 (0%) | 231 | 6.7% | 1.08 | 7 | 76% (3 months) | NR | 16.7 % | 4.5% | NR |
| Bhandari et al. | 300 | 109 (0%) | 177.5 | 5.7% | 1.2 | 6.9 | NR | NR | NR | NR | NR |
| Cathelineau et al. | 105 | 500 (6%) | 180 | 7.6% | 5.5 | 7 | NR | NR | 22.0 % | 11.7% | NR |
| Costello et al. | 122 | NR (3%) | NR | 15.6% | 2 | 8.4 | 82% (6 months) | NR | 16.3 % | NR | NR |
| Hu et al. | 322 | 250 (1.6%) | 186 | 14.6% | NR | NR | NR | NR | NR | NR | NR |
| Joseph et al. | 50 | 206 (0%) | 202 | 12.0% | NR | NR | 90% (3 months) | NR | 12.0 % | NR | 100% (5.3 months) |
| Menon et al. | 200 | 153 (0%) | 160 | 4.0% | 1.2 | 7 | 96% (6 months) | *64% > 60 38% < 60 | 6.0% | NR | 92% (6 months) |
| Patel et al. | 200 | 75 (0%) | 141 | 2.0% | 1.1 | 7.2 | 98% (12 months) | NR | 10.5 % | 5.7% | 95% (9.7 months) |
| Wolfram et al. | 81 | 300 (12%) | 250 | NR | NR | 14 | NR | NR | 22.2 % | 12.7% | NR |

EBL, estimated blood loss (cubic centimeters); Tx, transfusion rate; OR time, operative time (minutes); Compl., complication rate; LOS, length of stay (days); Catheter, length of catheterization (days); PMR, positive margin rate; BFS, biochemical-free survival; NR, not reported.

* Percentage of postoperative sexual intercourse in men less than 60 years and older than 60 years.

### Length of Hospitalization

One obvious advantage of minimally invasive surgery is decreased length of hospitalization. A quicker discharge from the hospital is appealing to many patients and may help to offset the increased cost associated with RLRP. The mean length of hospitalization is just over 24 h in most RLRP series from the U.S.[19,20,21]. Investigators have reported that more than 90% of patients can be safely discharged home in less than 24 h with one series reporting 5% of patients discharged home on the same day of surgery[20,21,39]. While reports of short hospital stay after RLRP are impressive, this may not be a great advantage over open surgery as early discharge after ORP is also feasible[40].

### Pain

Another traditional advantage of minimally invasive surgery has been less pain in the postoperative period. This has been well documented in urologic surgery for patients undergoing laparoscopic radical nephrectomy[41]. However, unlike open radical nephrectomy, the lower midline incision used for retropubic radical prostatectomy is not particularly painful by comparison. While some investigators have reported less postoperative pain after RLRP compared to ORP, this potential advantage of RLRP has been questioned[22]. Webster et al. prospectively assessed postoperative pain in patients undergoing RLRP and ORP using an identical pathway for pain control in both groups[42]. They found that total narcotic requirements did not significantly differ between the two groups and while the mean Likert pain score was lower in the RLRP group on the day of surgery (2.05 vs. 2.60), it was not significantly different on postoperative days 1 and 14. The authors attributed these findings to the minimal amount of postoperative pain in both groups. Therefore, it seems that postoperative pain does not appear to be as much of an advantage for RLRP in contrast to other minimally invasive surgical procedures.

### Length of Catheterization

Most surgeons perform a running urethral anastomosis under magnified visualization during RLRP. In contrast, during ORP, an interrupted anastomosis is performed under poorer visualization. This has led some surgeons to consider earlier catheter removal after RLRP. Unfortunately, the limiting factor for successful early catheter removal after radical prostatectomy appears to be high rates of urinary retention rather than the integrity of the anastomosis between urethra and bladder[43,44]. Most published series report catheterization times of approximately 7 days after RLRP (Table 2). At this time, it is questionable whether early catheter removal will be a significant advantage for RLRP over ORP.

### Complications

Perioperative complications associated with radical prostatectomy can be divided into intraoperative and postoperative complications. Intraoperative complications have been reported to occur in less than 1% of patients who undergo either ORP or RLRP[37,38]. The most common intraoperative complications include injury to the rectum, ureter, obturator nerve, and major vascular structures. There appears to be no difference in the incidence of these specific complications during RLRP vs. ORP. Postoperative complication rates also appear to be similar. ORP series have reported postoperative complication rates of 4.3–9% whereas early data from RLRP series suggest 1–12% of patients may have complications within 30 days of surgery[19,20,21,23,24,30,37,38]. The incidence of the more common postoperative complications including anastomotic stricture, thromboembolic events, and infection seem to be comparable between techniques.

Potential complications unique to the laparoscopic approach include abdominal wall bleeding or injury to intra-abdominal structures secondary to port placement, as well as hypercarbia secondary to pneumoperitoneum. Patients are also positioned differently during RLRP compared to ORP. Patients are in lithotomy during RLRP (rather than supine as for ORP) and special care is necessary to ensure proper positioning and padding to avoid neuropraxia. One well-known complication that can occur after ORP and pelvic lymphadenectomy is postoperative lymphocele. Theoretically, this complication would be less likely to occur after transperitoneal RLRP due to the intraperitoneal reabsoprtion of lymph fluid.

### OTHER ISSUES UNIQUE TO RLRP

#### Preoperative Counseling

A few specific issues should be discussed with patients prior to RLRP surgery. As with all laparoscopic surgery, patients should be informed of the potential need for conversion to an open surgical procedure. Patients should understand that conversion to an open procedure should not be considered a complication, but rather should be judiciously performed when the surgeon feels a suboptimal procedure will result by continuing robotically. Ultimately, the goal is to perform the best and safest operation for the patient whether laparoscopically or open. During the early learning phase of RLRP, surgeons should maintain a low threshold for converting to an open procedure in the best interest of their patients.

Another issue worth discussion is the real risk of mechanical failure of the robotic system. The incidence of robotic malfunction was reported to be 0.6% by Hu et al.[37]. At Virginia Mason Medical Center, we found the mechanical failure rate to be 4.6% in our first 130 cases (submitted abstract). The patient has a few options when malfunction occurs prior to anesthesia induction. They may proceed with a conventional laparoscopic approach, open surgery, or reschedule the procedure at a time when the system is functional. Each of these options is associated with psychological, financial, and logistic burdens for the patient, physicians, and hospital involved. Complete reliance on technology is a new issue in major urologic surgery. For most surgical procedures, duplicate supplies of critical surgical instruments are available. However, most institutions have only one available robotic system due to the high cost. We recommend patients be counseled preoperatively that malfunction of the robotic system during the course of a procedure can occur, resulting in conversion to a conventional laparoscopic procedure or an ORP. We also question all patients preoperatively so they can express their desire as to how they would like to proceed if the system becomes nonoperational between the induction of anesthesia and start of the surgical procedure.

#### Training

While it is generally accepted that open radical prostatectomists can learn to perform RLRP quicker than conventional LRP, the actual learning curve for RLRP is controversial. Some have concluded that the learning curve may be anywhere from 10–25 cases[20,45]. However, others with extensive experience with ORP have felt the learning curve requires more than 250 cases[46]. This wide variability is likely due to different definitions of what constitutes achieving expertise.

How to best initiate a RLRP program is currently a topic of discussion. At institutions with no RLRP experience, the combination of a surgeon with extensive laparoscopic experience and a surgeon with extensive ORP experience appears to be effective when beginning a program[20,45,46,47]. Other surgeons have chosen to implement their program after first attending training courses. Still others have sought fellowship training as a way to learn RLRP technique.

The most efficient method to teach the RLRP technique has not been established. In contrast to open surgery where teaching surgeons can closely monitor trainee performance in a controlled environment, RLRP allows only one surgeon to work at the operative console at a time. To circumvent this problem, we have implemented a teaching program that divides the procedure into stages. This method allows the trainee surgeon to increase their console operating time gradually and perform the more challenging technical aspects only after mastering the simpler portions of the procedure. It also maintains efficiency since only a part of the surgery is subjected to a learning curve, while experienced surgeons perform the remainder of the operation. We have found this method useful in training attending urologists, fellows, and residents with minimal effect on outcomes, complication rates, and operative time (submitted abstract).

#### Cost

The most apparent disadvantage of RLRP is the associated cost. The initial cost of the daVinci system approaches $1,400,000. Maintenance fees for technical support are more than $100,000 annually. There are also added intraoperative expenses due to the specialized instrumentation and lost OR time from longer setup and turnover times between cases. A cost comparison study estimated that RLRP was more expensive than LRP and both were more expensive than retropubic radical prostatectomy[48]. Using a cost model analysis, Scales et al. determined that cost is dependent on case volume [49]. These authors predicted RLRP and retropubic radical prostatectomy would be cost competitive at high volume centers. In the absence of true proven advantages for patients, higher cost should be considered a disadvantage of RLRP compared to open surgery.

### CONCLUSIONS

RLRP is now widely available to patients. More than 8,000 RLRPs were performed in the U.S. in 2004 with twice that many cases estimated to have been performed in 2005[50]. RLRP has been publicized as a minimally invasive surgical treatment for prostate cancer with theoretical benefits over traditional ORP techniques. Currently, there are no prospective, randomized trials comparing RLRP against ORP, and no long-term data to support these claims. However, data are beginning to emerge as investigators seek to study this technology objectively to determine its true advantages and role in prostate cancer treatment.

The current available data suggest that RLRP appears to be at least equivalent to ORP in terms cancer control, urinary control, and erectile function outcomes. It is also becoming apparent that the traditional benefits of minimally invasive surgery such as decreased postoperative pain, shorter hospital stay, and quicker convalescence are not as significant for radical prostatectomy in comparison to other procedures. There are promising data, however, that there may be advantages for RLRP in terms of improved postoperative potency and possibly oncologic outcomes. Presently, these advantages remain theoretical and future studies will determine the role that robotic-assisted surgery will have in the treatment of men with prostate cancer.
